# Preliminary In Vitro Evaluation of Silver, Copper and Gold Nanoparticles as New Antimicrobials for Pathogens That Induce Bovine Locomotion Disorders

**DOI:** 10.3390/ijms25179494

**Published:** 2024-08-31

**Authors:** Aleksandra Kalińska, Cezary Wawryło, Wiktoria Tlatlik, Marcin Gołębiewski, Magdalena Kot, Agata Lange, Sławomir Jaworski

**Affiliations:** 1 Animal Breeding Department, Warsaw University of Life Sciences, 02-786 Warszawa, Poland; 2 Department of Nanobiotechnology, Warsaw University of Life Sciences, 02-786 Warszawa, Poland

**Keywords:** dairy cows, locomotor disorders, metal nanoparticles, antibacterial activity

## Abstract

Lameness is a crucial problem in dairy farming. It worsens the welfare of cattle, reduces the milk yield, and causes economic losses. The etiology of lameness is varied and the cattle’s condition may be infectious or non-infectious. The aim of this research was to analyze the biocidal properties of silver (AgNPs), gold (AuNPs), and copper (CuNPs) nanoparticles against bacteria causing lameness in cattle. The isolated pathogens used were *Aerococcus viridans*, *Corynebacterium freneyi*, *Corynebacterium xerosis*, and *Trueperella pyogenes*. The tested concentrations of nanoparticles were 50, 25, 12.5, 6.25, 3.125, and 1.56 mg/L. The methods used included the isolation of pathogens using standard microbiological procedures and their identification using mass spectrometry, physicochemical analysis, transmission electron microscopy, and cytotoxicity tests. Studies have shown that AgNPs at 3.125 and 1.56 mg/L concentrations, and CuNPs at 25 and 12.5 mg/L concentrations, have strong biocidal properties, while AuNPs have the weakest antimicrobial properties. The very limited number of in vivo studies focusing on lameness prevention in cattle indicate that new solutions need to be developed. However, further studies are necessary to evaluate if nanoparticles (NPs) may, in the future, become components of innovative biocides used to prevent lameness in dairy cattle.

## 1. Introduction

Cattle lameness is a crucial problem that not only negatively affects animal health but also causes high economic losses. Most often, the condition is caused by hoof lesions, which can either be infectious (e.g., digital dermatitis or interdigital dermatitis) or non-infectious (e.g., white line disease or interdigital hyperplasia) [1]. The condition reduces the milk yield, leads to weight loss, decreases reproductive rates, and often involves animal culling. In addition to the mentioned effects, the advanced stages of lameness may cause fever, swelling, and limited mobility. Cows with lameness have lower feed intake because of the condition of the limb, which limits their ability to stand and reach the feed [2]. In subclinical cases of lameness, the disease is often not observed, but the cow’s milk yield may begin to decline [3], which indicates the importance of properly treating the clinical stages of this disease but also the significance of early detection.

Lameness caused by infectious agents can be easily transferred to other animals in the herd [4], and the identification of infectious microbes is difficult because of the multifactorial etiology [5]. The bacteria most often isolated from cows with lameness are *Treponema*, *Bacteroides*, *Mycoplasma* spp., *Campylobacter* spp., *Fusobacterium necrophorum*, and *Borrelia burgdorferi* [6]. Globally, the problem of lameness affects between 17% and 35% of dairy cattle. The disease occurs more frequently in intensive breeding systems [7]. The available studies have revealed that lameness occurs in 72% of European dairy herds [1], which suggests that the problem is significant in modern dairy breeding. In infectious cases, the most common method of treating lameness is the use of antibiotics [8], but in the long term, this increases bacterial resistance to antibiotics [9]. Additionally, the treated animal is subject to a withdrawal period, which results in economic losses. After mastitis, lameness is the second most important factor causing losses in the dairy industry [10].

Due to increasing antibiotic resistance, alternative biocidal agents are constantly being sought. One of the alternatives to antibiotics could involve the use of nanotechnology, including metal nanoparticles (NPs). Their small size and selectivity in relation to bacteria have made certain types of metal NPs effective methods for fighting pathogens [11]. Moreover, it is hypothesized that metal NPs with antibacterial properties can be used to reduce the evolution of more resistant bacteria. This is due to the NPs’ mechanism of action, which targets many biomolecules at the same time, making it difficult for bacteria to develop effective resistance mechanisms [12].

There are no reports in the literature on the association of the pathogens *Aerococcus viridans*, *Corynebacterium freneyi*, *Corynebacterium xerosis*, and *Trueperella pyogenes* with lameness in dairy cattle. Often, information about these bacteria can be found in relation to diseases in swine [13] and sheep [14]. There are studies in which *Aerococcus viridans* [15], *Trueperella pyogenes* [16], and *Corynebacterium* spp. [17] are indicated as the etiological factors for mastitis. While research on *Corynebacterium xerosis* can be found [18], there are very few publications on *Corynebacterium freneyi*. Moreover, in the world of science, there is a lack of research involving these bacteria and nanobiotechnology, which is why there are no reports on the effect of using nanomaterials on the discussed pathogens that cause cattle lameness—this highlights the need for new research in this area.

The aim of this study was to estimate the antimicrobial effect of AgNPs, CuNPs, and AuNPs on four pathogens isolated from cows with clinical locomotor disorders: *Aerococcus viridans*, *Corynebacterium freneyi*, *Corynebacterium xerosis*, and *Trueperella pyogenes*.

## 2. Results

### 2.1. Identification of the Isolated Bacteria

The results of the identification performed using the MALDI-TOF MS (Bruker, Poznań, Poland) are presented in Table 1. A score value of >2.0 demonstrates the identification of the pathogen to a specific strain.

### 2.2. Determination of the NPs’ Morphology

**Figure 1:**
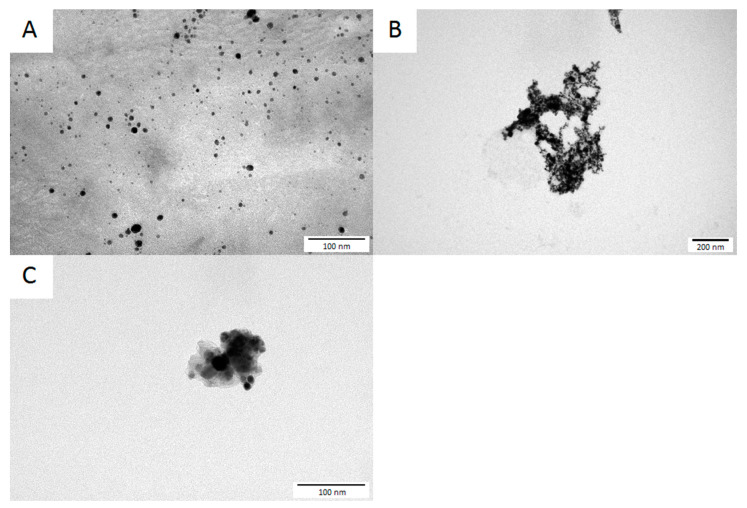
Transmission electron microscopy images of the studied NPs: (**A**)—AgNPs, (**B**)—AuNPs, and (**C**)—CuNPs.

The morphology of the NPs (presented in Figure 1) was analyzed based on images taken by transmission electron microscopy (TEM). All of the tested NPs had approximately spherical morphology and a size range of approximately 1–20 nm (AgNPs), 5–40 nm (AuNPs), and 5–25 nm (CuNPs).

### 2.3. Determination of the Physicochemical Properties of NPs

**Figure 2:**
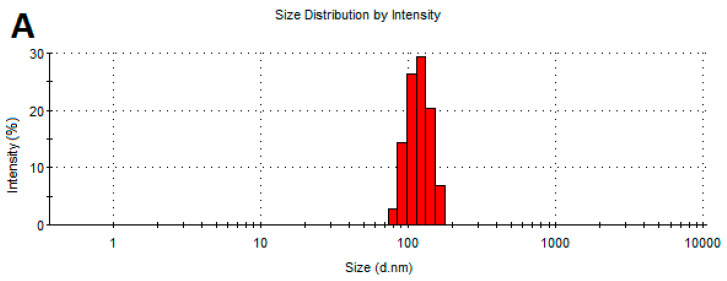

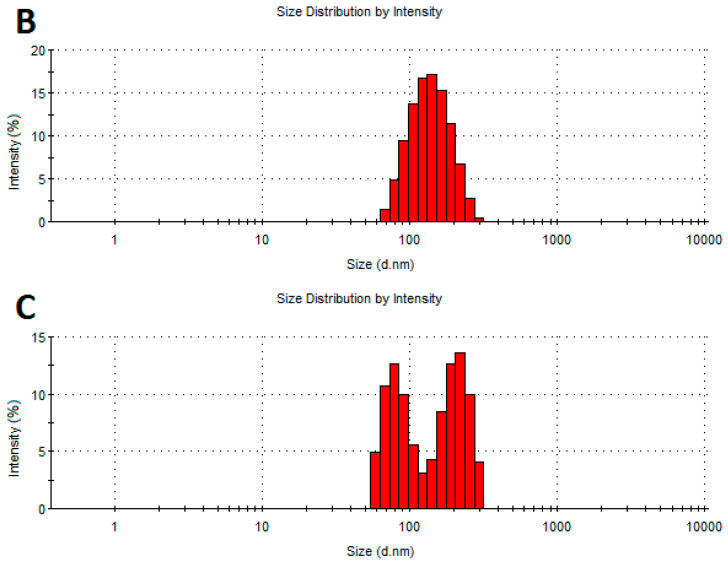
Size distribution of the NPs: (**A**)—AgNPs, (**B**)—AuNPs, and (**C**)—CuNPs.

Figure 2 shows the size distribution of the NPs. All the studied NPs showed a tendency to agglomerate. An analysis of the size distribution of the AgNPs, CuNPs, and AuNPs showed no significant variation in size. The size of most AgNP and AuNP agglomerates was between 100 and 200 nm. The CuNP agglomerates formed two main groups: their sizes were around 60–90 nm and 200–300 nm.

**Figure 3:**
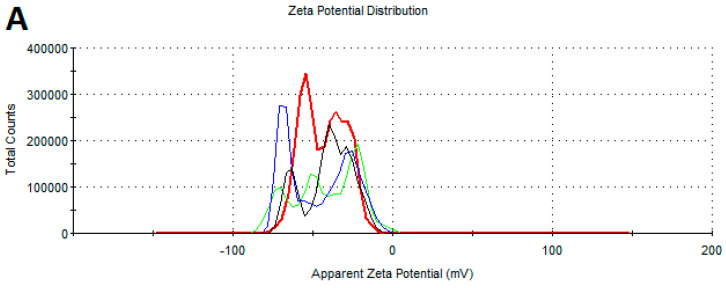

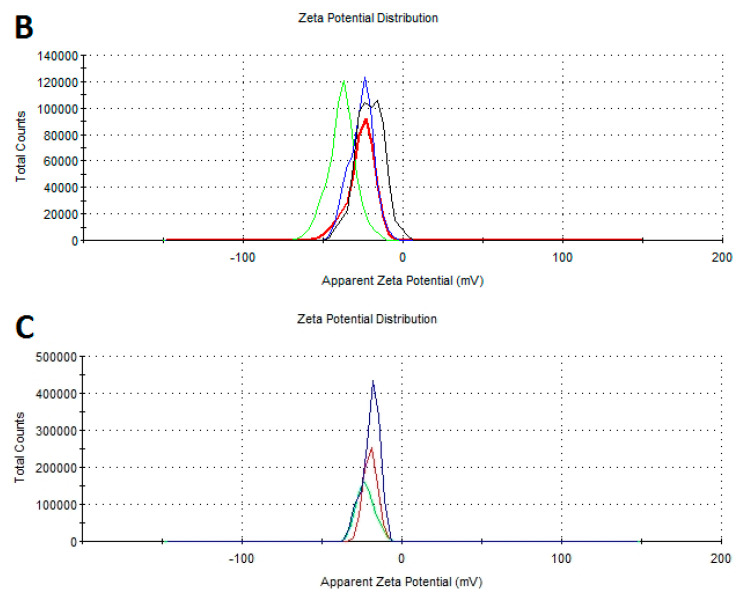
Zeta potential distribution of the NPs: (**A**)—AgNPs, (**B**)—AuNPs, and (**C**)—CuNPs.

Table 2 shows the average zeta potential values from the three measurements taken for all the tested NPs. The zeta potential indicates the stability of the nanomaterials. All the tested NPs showed negative zeta potential values. The most negative value was observed for the AgNPs, while the zeta potential of the CuNPs and AuNPs was at a similar level. Figure 3 shows the zeta potential distribution of the NPs.

### 2.4. Bacterial Viability Analysis

**Figure 4:**
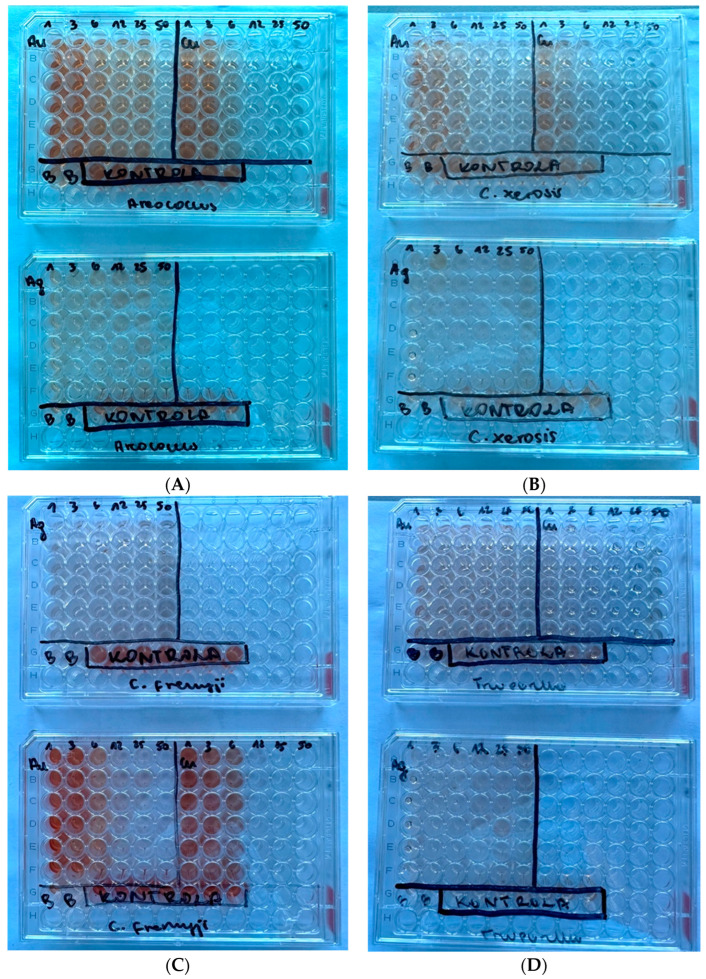
Results of the XTT assay for each examined strain: (**A**) *Aerococcus viridans*, (**B**) *Corynebacterium xerosis*, (**C**) *Corynebacterium freneyi*, and (**D**) *Trueperella pyogenes*.

**Figure 5:**
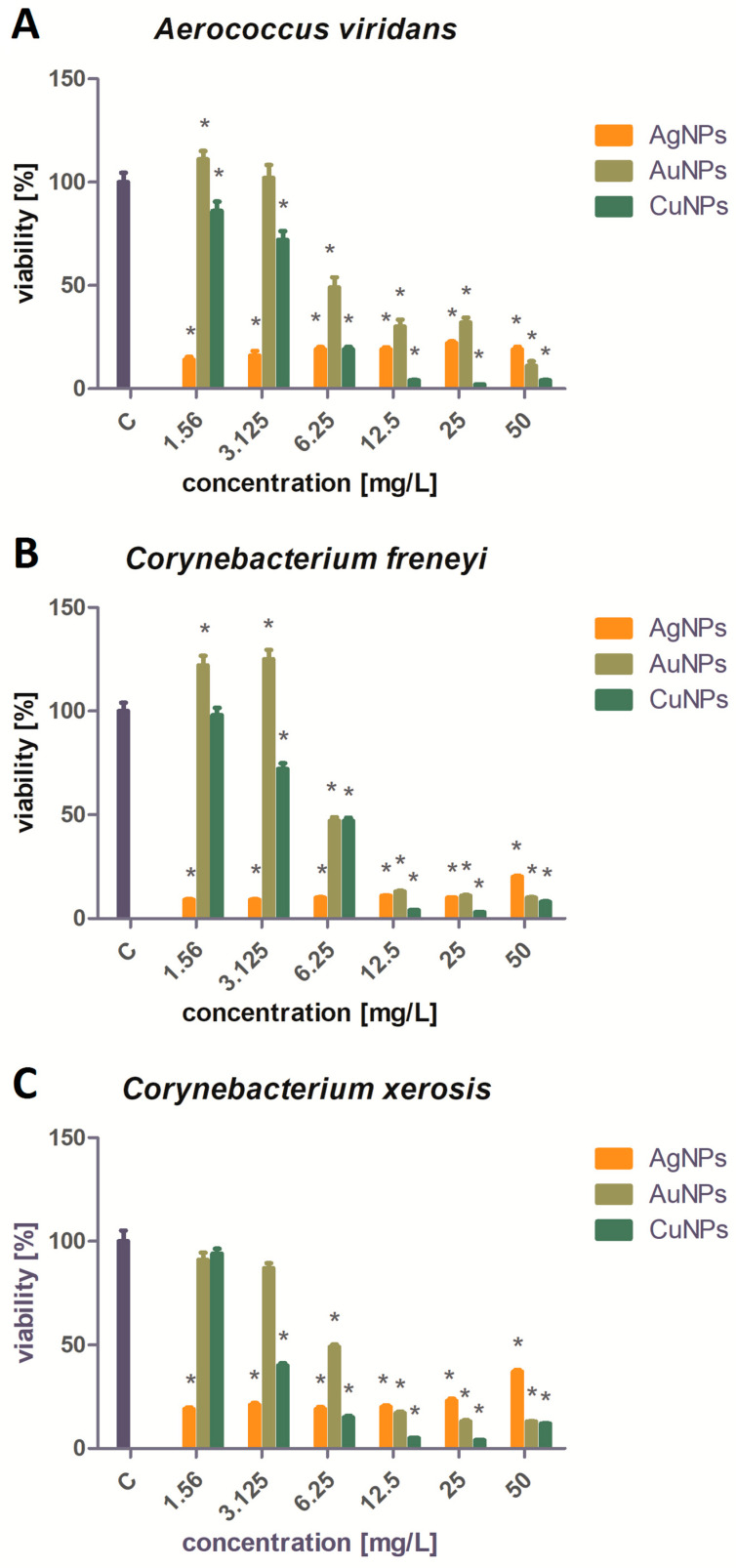

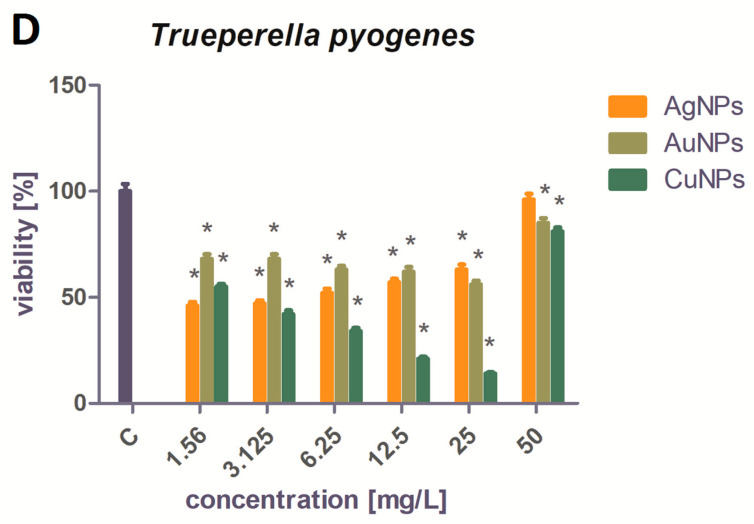
Viability analysis of the tested pathogens treated with Ag, Au, and Cu NPs (%): (**A**)—*Aerococcus viridans*, (**B**)—*Corynebacterium freneyi*, (**C**)—*Corynebacterium xerosis*, and (**D**)—*Trueperella pyogenes*. * Significant differences in comparison to control (*p* < 0.05).

The results of the conducted XTT assay after incubation with NPs are presented in Figure 4 for each strain. The percentage viability of the bacteria is presented in Figure 5. For all the pathogens except *Trueperella pyogenes*, the strongest biocidal effect of all the tested NPs was observed after using the three highest NP concentrations: 50 mg/L, 25 mg/L, and 12.5 mg/L. An AuNP concentration of 6.25 mg/L was associated with a decrease in bacterial viability of up to about 40–50%. The use of lower concentrations of AuNPs did not significantly reduce the bacterial viability. The AgNPs showed strong antibacterial activity at every concentration tested, reducing the viability by 80–90%. The weakest biocidal activity, not counting the results obtained for *Trueperella pyogenes*, was shown by the AgNPs at a concentration of 50 mg/L against *Corynebacterium xerosis*. At the three highest concentrations tested, the CuNPs showed the strongest biocidal activity out of all the tested NPs.

*Trueperella pyogenes* showed greater resistance to the biocidal effects of the NPs than the other three microorganisms. Only the AgNP 50 mg/L concentration did not significantly reduce the viability of the bacteria. The viability of *Trueperella pyogenes* bacteria treated with AgNPs and AuNPs at concentrations of 25, 12.5, 6.25, 3.125, and 1.56 mg/L ranged from 47% to 68%. The strongest bactericidal activity against *Trueperella pyogenes* was observed after the application of CuNPs. The most effective concentration of CuNPs (25 mg/L) reduced the bacterial viability by 86%.

## 3. Discussion

Antibiotic resistance in bacteria is a problem that not only affects human health but also animal welfare. It is estimated that 30–90% of the antibiotics used in veterinary treatment are excreted by animals in an unchanged form or as an active metabolite. Antibiotics that enter the environment allow bacteria to develop resistance, making them a greater threat to animal health [19]. One of the alternatives to antibiotics is nanobiotechnology and the use of NPs. Research using metal NPs has shown particularly high effectiveness in combating bacteria [11].

Metal NPs are known for not binding to a specific bacterial receptor, making it more difficult for pathogens to develop effective resistance. The antibacterial properties of metal NPs have already been proven against both Gram-negative and Gram-positive bacteria [20]. The exact mechanism utilized by the NPs in their antibacterial action is not yet known, but there are theories regarding the possible mechanisms. The modes of action are as follows: overproduction of reactive oxygen species by bacteria, damage to the pathogen’s cell membrane, accumulation of metal ions within membranes, electrostatic attraction between metal NPs and the microorganism’s cell membrane, and the inhibition of bacterial enzymes and proteins by increasing the production of hydrogen peroxide [21]. Silver and copper NPs have proven to have strong antibacterial properties across a wide range of concentrations [22]. The antibacterial activity of gold NPs is disputed. Most studies have reported that AuNPs only have weak biocidal properties and these only occur at high concentrations [23], but combining them with biomolecules or contamination may reduce the viability of microorganisms and even enhance the biocidal effects of the substances with which they are conjugated [24].

The ability to compare the obtained results to those of other authors is limited, because studies on isolated bacteria are not widely described in the literature. The identified bacteria have also not been linked to bovine lameness. The available literature on the results obtained by other authors is shown in Table 3—no results were found for the study of the antibacterial effect of NPs on *C. freneyi*. Therefore, the authors compared the results of the analyses of the antimicrobial properties of the tested NPs against Gram-positive bacteria, as all the tested strains were Gram-positive.

According to Kalińska et al., Gram-positive bacteria are less sensitive to the influence of AgNPs than Gram-negative bacteria, while CuNPs are more toxic to Gram-positive bacteria [30]. This was partially reflected in the results, as the CuNPs in higher concentrations—especially 50, 25, and 12.5 mg/L—showed a stronger bactericidal effect against all the tested bacteria. However, the CuNP concentration of 6.25 mg/L turned out to be less effective against *C. freneyi*, as it reduced the viability of the bacteria by only 53%, whereas the AgNPs reduced the viability by 90%. The AgNPs were more universal because they were effective at a similar level regardless of the concentration used, while lower concentrations of the CuNPs (3.125 and 1.56 mg/L) were much less effective in reducing the viability of pathogens. The higher effectiveness of AgNPs at lower concentrations could be the result of greater homogeneity and stability, which was demonstrated in the analysis of the physicochemical properties of the NPs as well as in studies by Mohamad Kasim et al. [31]. Another reason for the high effectiveness of the CuNPs and AgNPs may be their small size. In the current study, the AgNPs had the smallest size and they were also observed to result in the most uniform, high bactericidal activity—at similar activity levels across different concentrations. This is due to the fact that the larger surface-area-to-volume ratio at smaller sizes results in a faster release of ions, which are responsible for the NPs’ toxicity to bacteria [32].

As shown in a study by Shamaila et al., low concentrations of AuNPs did not demonstrate biocidal properties against Gram-positive bacteria [33]. Moreover, for *A. viridans* and *C. freneyi*, results similar to a study by Kot et al. were observed. Low concentrations of AuNPs (1.56 and 3.125 mg/L) promoted bacterial growth [34]. In contrast to current reports by Allahverdiyev et al. [35] about AuNPs’ lack of antimicrobial properties, all the tested strains turned out to be sensitive to high concentrations of AuNPs, except *Trueperella pyogenes*. According to Dasari et al. [36], AuNPs do not inhibit bacterial growth, but Au(I) and Au(III) ions have strong antibacterial properties, which may suggest that the gold used in the current research was in the form of ions. A disturbing outcome seen in the results was the resistance of *Trueperella pyogenes* to nanomaterials—especially to AgNPs—as in research by Rezanejad et al., a concentration of 1 mg/L reduced the bacterial viability by over 90% [37], but in the current study, a concentration of 1.56 mg/L only reduced the bacterial viability by 54%. The obtained results, and the fact that the literature on the tested pathogens is limited, highlight the need for further research on this topic.

An important issue in terms of the use of NPs is their safety to animals and the environment. The toxicity of nanomaterials varies depending on their concentration and how they are introduced into animals. The strongest toxicity is induced by injection and oral administration of NPs [38], but the toxicity of NPs can be reduced by external application. The presence of AgNPs and AuNPs in the body can induce toxic effects against the liver, thyroid or fertility, but at low concentrations, these nanoparticles do not cause changes in the organs [39]. However, the uncontrolled entry of NPs into the environment can have negative consequences for the aquatic environment, and the bioaccumulation of NPs in aquatic organisms can amplify along the food chain and cause changes in these organisms [40]. Research on the safety of NP use is a key issue and must be conducted on a par with research on the use of nanomaterials.

## 4. Materials and Methods

### 4.1. Isolated Bacteria Cultures

**Figure 6:**
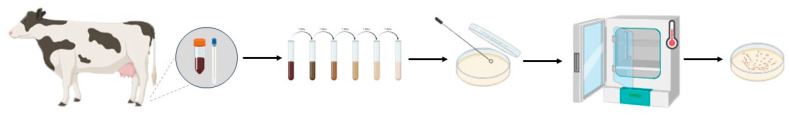
Methodological diagram illustrating the collection of biological material (swabs, pus and tissue samples) and its further processing (created with BioRender.com).

Swabs, pus and tissue samples were collected during hoof correction (standard zootechnical procedure) from the limbs of cattle diagnosed with lameness and placed in sterile boxes and transported to a laboratory at the Department of Animal Breeding of the Warsaw University of Life Sciences, as presented in Figure 6. The collected samples were placed in sterile 0.9% NaCl at eight dilutions up to 10^−8^ in order to prepare inoculations. Bacteria cultures were cultivated using Columbia Blood Agar with 5% KB (Biomaxima, Lublin, Poland) under aerobic and anaerobic conditions at 37 °C for 24–72 h. In order to create the anaerobic culture conditions for the microbiological cultures, GENbag (bioMérieux, Warsaw, Poland) microorganism identification was performed using a MALDI-TOF mass spectrometer (MS) (Bruker, Poznań, Poland), which enables the identification of bacteria using mass spectrometry and is based on the m/z ratio of ions in the tested sample to the ratio in the NCBI (National Center for Biotechnology Information) database. Each NCBI strain has a unique number connected to a specific strain.

### 4.2. The Morphology of NPs

Commercially available hydrocolloidal AgNPs, CuNPs, and AuNPs were used in this study (Nano-Tech, Warsaw, Poland). The NPs were synthesized using physical methods. According to the manufacturer, the company has an innovative patented method for physically obtaining non-ionic precious and semi-precious metal NPs. The laboratory disintegration of pure metals into particles results in the creation of NPs with sizes from a few to several nanometers.

To determine their morphology, the NPs were viewed using a JEM-1220 transmission electron microscope (Jeol, Tokyo, Japan) at 80 mV. Hydrocolloids of NPs were sonicated for three minutes, then applied to formvar-coated copper grids (Agar Scientific, Stansted, UK) and left to air-dry.

### 4.3. The Physicochemical Properties of NPs

Zeta potential measurements and size distribution measurements were performed using a Zetasizer Nano-ZS90 analyzer (Malvern Instruments, Worcestershire, UK). The average size of the NPs was measured by dynamic light scattering (DLS).

### 4.4. Bacterial Viability

**Figure 7:**
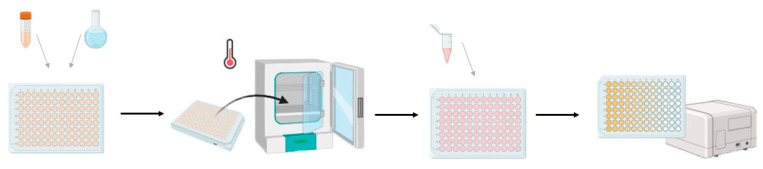
Methodological diagram illustrating the bacterial viability analysis using an XTT assay (created with BioRender.com).

A bacterial viability test was carried out according to the diagram in Figure 7. Bacterial suspensions with an optical density of 0.5 on the McFarland scale were prepared with sterile 0.9% NaCl. The groups for the 50 mg/L bacterial concentration were prepared separately. The suspensions were pipetted into a 96-well plate at a volume of 50 μL per well. Nanoparticle hydrocolloids were sonicated for three minutes and then applied to the plate at 50 μL per well. The concentrations of NPs in the wells were as follows: 50, 25, 12.5, 6.25, 3.125, and 1.56 mg/L. The bacteria were incubated for 24 h at 37 °C. After 24 h, 20 μL of XTT reagent (Sigma-Aldrich Cell Proliferation Kit II, Merck, Darmstadt, Germany) was added to each well. The bacteria were incubated for the next 24 h at 37 °C and then the absorbance was read using an Infinite M200 microplate reader (Tecan, Morrisville, NC, USA) at a wavelength of 450 nm and a reference of 690 nm.

### 4.5. Statistical Analysis

The results of the bacterial viability analysis were statistically processed using univariate analysis of variance (ANOVA) and the Tukey’s post hoc test using GraphPad Prism 8 software. Differences with a *p*-value of <0.05 were considered statistically significant.

## 5. Conclusions

The conducted research has shown that AgNPs, AuNPs, and CuNPs influence the viability of the isolated pathogens. This effect varies depending on the microorganism and the concentration of the NPs. The CuNPs had the strongest biocidal effect against all the tested bacteria. The most resistant strain was *Trueperella pyogenes*. The strongest bactericidal effect against this strain was observed for the CuNPs at a concentration of 25 mg/L. The AgNPs showed strong antibacterial activity against *Aerococcus viridans*, *Corynebacterium freneyi*, and *Corynebacterium xerosis*; the lowest viability was observed for concentrations of 1.56 and 3.125 mg/L against *Corynebacterium freneyi*. The AuNPs showed the strongest bactericidal effect at a concentration of 50 mg/L—the exception being *Trueperella pyogenes*. No bactericidal effect was observed for the AuNP concentrations of 1.56 or 3.125 mg/L. The lowest concentration of CuNPs (1.56 mg/L) also did not significantly reduce the viability of half of the tested bacteria. The results indicate that some of the tested concentrations and types of NPs can be used to treat lameness in cattle, but much depends on the etiology of the disease. Additionally, further research is necessary to expand knowledge not only about the bactericidal properties of the tested NPs against pathogens causing lameness but also the safety of using these nanomaterials in cattle.

## Figures and Tables

**Table 1:** Pathogens identified using the MALDI-TOF MS, along with the resulting score value and the NCBI identifier.

Matched Pattern	Score Value	NCBI Identifier
*Aerococcus viridans*	2.10	1377
*Corynebacterium freneyi*	2.25	134,034
*Corynebacterium xerosis*	2.14	1725
*Trueperella pyogenes*	2.02	1661

**Table 2:** Average zeta potential of the NPs (mV).

	AgNPs	AuNPs	CuNPs
Zeta potential	−48.2	−21.6	−22.4

**Table 3:** Summary of recent studies on the antibacterial activity of AgNPs and CuNPs against the tested pathogens [25,26,27,28,29].

Pathogen	Type of Study	NPs Used	Results	Source
*A. viridans*	Agar well diffusion method	AgNPs	Mean zone of inhibition was 22 ± 0.41 mm.	[25]
		CuNPs	Mean zone of inhibition was 10 mm and 13 mm.	[26]
*C. xerosis*	Agar well diffusion method	AgNPs	Mean zone of inhibition was 13.6 ± 0.50 mm.	[27]
	In vitro	rGO/CuNPs	Bacterial viability was reduced by ~99.6%.	[28]
*T. pyogenes*	MIC	AgNPs	Minimum inhibitory concentration was 1.0 mg/L.	[29]
	MBC		Minimum bactericidal concentration was 1.5 mg/L.	
	In vitro		Inhibition of biofilm formation was reduced by over 90%.	
			The level of lactate dehydrogenase was four-fold higher compared to the control.	
			The level of reactive oxygen species was two-fold higher compared to the control.	

## Data Availability

Data are available on request from the authors.

## References

[1] Van Nuffel A., Zwertvaegher I., Pluym L., Van Weyenberg S., Thorup V.M., Pastell M., Sonck B., Saeys W. (2015). Lameness detection in dairy cows: Part 1. How to distinguish between non-lame and lame cows based on differences in locomotion or behavior. Animals.

[2] Afonso J.S., Bruce M., Keating P., Raboisson D., Clough H., Oikonomou G., Rushton J. (2020). Profiling detection and classification of lameness methods in British dairy cattle research: A systematic review and meta-analysis. Front. Vet. Sci..

[3] Langova L., Novotna I., Nemcova P., Machacek M., Havlicek Z., Zemanova M., Chrast V. (2020). Impact of nutrients on the hoof health in cattle. Animals.

[4] Ramanoon S.Z., Sadiq M.B., Shaik Mossadeq W.M., Mansor R., Syed-Hussain S.S. (2018). The impact of lameness on dairy cattle welfare: Growing need for objective methods of detecting lame cows and assessment of associated pain. Anim. Welf..

[5] Palmer M.A., O’Connell N.E. (2015). Digital dermatitis in dairy cows: A review of risk factors and potential sources of between-animal variation in susceptibility. Animals.

[6] Zinicola M., Lima F., Lima S., Machado V., Gomez M., Döpfer D., Guard C., Bicalho R. (2015). Altered microbiomes in bovine digital dermatitis lesions, and the gut as a pathogen reservoir. PLoS ONE.

[7] Blackie N., Maclaurin L. (2019). Influence of lameness on the lying behaviour of zero-grazed lactating jersey dairy cattle housed in straw yards. Animals.

[8] Coetzee J.F., Shearer J.K., Stock M.L., Kleinhenz M.D., van Amstel S.R. (2017). An update on the assessment and management of pain associated with lameness in cattle. Vet. Clin. Food Anim. Pract..

[9] Saleem M., Deters B., de la Bastide A., Korzen M. (2019). Antibiotics overuse and bacterial resistance. Ann. Microbiol. Res..

[10] Garvey M. (2022). Lameness in dairy cow herds: Disease aetiology, prevention and management. Dairy.

[11] Mubeen B., Ansar A.N., Rasool R., Ullah I., Imam S.S., Alshehri S., Ghoneim M.M., Alzarea S.I., Nadeem M.S., Kazmi I. (2021). Nanotechnology as a novel approach in combating microbes providing an alternative to antibiotics. Antibiotics.

[12] Slavin Y.N., Asnis J., Hńfeli U.O., Bach H. (2017). Metal nanoparticles: Understanding the mechanisms behind antibacterial activity. J. Nanobiotechnol..

[13] Monteiro M.S., Matias D.N., Poor A.P., Dutra M.C., Moreno L.Z., Parra B.M., Silva A.P.S., Matajira C.E.C., Gomes V.T.M., Barbosa M.R.F. (2022). Causes of sow mortality and risks to post-mortem findings in a Brazilian intensive swine production system. Animals.

[14] Queiroga M.C. (2017). Prevalence and aetiology of sheep mastitis in Alentejo region of Portugal. Small Rumin. Res..

[15] Sun M., Gao J., Ali T., Yu D., Zhang S., Khan S.U., Fanning S., Han B. (2017). Characteristics of Aerococcus viridans isolated from bovine subclinical mastitis and its effect on milk SCC, yield, and composition. Trop. Anim. Health Prod..

[16] Rzewuska M., Kwiecień E., Chrobak-Chmiel D., Kizerwetter-Świda M., Stefańska I., Gieryńska M. (2019). Pathogenicity and virulence of *Trueperella pyogenes*: A review. Int. J. Mol. Sci..

[17] Dobinson H.C., Anderson T.P., Chambers S.T., Doogue M.P., Seaward L., Werno A.M. (2015). Antimicrobial treatment options for granulomatous mastitis caused by Corynebacterium species. J. Clin. Microbiol..

[18] Hernández-León F., Acosta-Dibarrat J., Vázquez-Chagoyán J.C., Rosas P.F., de Oca-Jiménez R.M. (2016). Identification and molecular characterization of Corynebacterium xerosis isolated from a sheep cutaneous abscess: First case report in Mexico. BMC Res. Notes.

[19] Huygens J., Daeseleire E., Mahillon J., Van Elst D., Decrop J., Meirlaen J., Dewulf J., Heyndrickx M., Rasschaert G. (2021). Presence of antibiotic residues and antibiotic resistant bacteria in cattle manure intended for fertilization of agricultural fields: A one health perspective. Antibiotics.

[20] Sánchez-López E., Gomes D., Esteruelas G., Bonilla L., Lopez-Machado A.L., Galindo R., Cano A., Espina M., Ettcheto M., Camins A. (2020). Metal-based nanoparticles as antimicrobial agents: An overview. Nanomaterials.

[21] Nisar P., Ali N., Rahman L., Ali M., Shinwari Z.K. (2019). Antimicrobial activities of biologically synthesized metal nanoparticles: An insight into the mechanism of action. JBIC J. Biol. Inorg. Chem..

[22] Fan X., Yahia L.H., Sacher E. (2021). Antimicrobial properties of the Ag, Cu nanoparticle system. Biology.

[23] Zhang Y., Shareena Dasari T.P., Deng H., Yu H. (2015). Antimicrobial activity of gold nanoparticles and ionic gold. J. Environ. Sci. Health.

[24] Tao C. (2018). Antimicrobial activity and toxicity of gold nanoparticles: Research progress, challenges and prospects. Lett. Appl. Microbiol..

[25] Hamouda R.A., Makharita R.R., Qarabai F.A., Shahabuddin F.S., Saddiq A.A., Bahammam L.A., El-Far S.W., Bukhari M.A., Elaidarous M.A., Abdella A. (2023). Antibacterial Activities of Ag/Cellulose Nanocomposites Derived from Marine Environment Algae against Bacterial Tooth Decay. Microorganisms.

[26] Román L., Castro F., Maúrtua D., Condori C., Vivas D., Bianchi A.E., Paraguay-Delgado F., Solis J.L., Gómez M.M. (2017). Nanopartículas de CuO y su propiedad antimicrobiana en cepas intrahospitalarias. Rev. Colomb. De Química.

[27] Siddiqi K.S., Rashid M., Rahman A., Tajuddin, Husen A., Rehman S. (2018). Biogenic fabrication and characterization of silver nanoparticles using aqueous-ethanolic extract of lichen (Usnea longissima) and their antimicrobial activity. Biomater. Res..

[28] Kim J., Kang S.H., Choi Y., Lee W., Kim N., Tanaka M., Kang S.H., Choi J. (2023). Antibacterial and biofilm-inhibiting cotton fabrics decorated with copper nanoparticles grown on graphene nanosheets. Sci. Rep..

[29] Gurunathan S., Choi Y.J., Kim J.H. (2018). Antibacterial efficacy of silver nanoparticles on endometritis caused by Prevotella melaninogenica and Arcanobacterum pyogenes in dairy cattle. Int. J. Mol. Sci..

[30] Kalińska A., Gołębiewski M., Wójcik A. (2017). Mastitis pathogens in dairy cattle—A review. World Sci. News.

[31] Mohamad Kasim A.S., Ariff A.B., Mohamad R., Wong F.W.F. (2020). Interrelations of synthesis method, polyethylene glycol coating, physico-chemical characteristics, and antimicrobial activity of silver nanoparticles. Nanomaterials.

[32] Kim J.S., Kuk E., Yu K.N., Kim J.H., Park S.J., Lee H.J., Kim S.H., Park Y.K., Park Y.H., Hwang C.Y. (2007). Antimicrobial effects of silver nanoparticles. Nanomed. Nanotechnol. Biol. Med..

[33] Shamaila S., Zafar N., Riaz S., Sharif R., Nazir J., Naseem S. (2016). Gold nanoparticles: An efficient antimicrobial agent against enteric bacterial human pathogen. Nanomaterials.

[34] Kot M., Kalińska A., Jaworski S., Wierzbicki M., Smulski S., Gołębiewski M. (2023). In vitro studies of nanoparticles as a potentially new antimicrobial agent for the prevention and treatment of lameness and digital dermatitis in cattle. Int. J. Mol. Sci..

[35] Allahverdiyev A.M., Kon K.V., Abamor E.S., Bagirova M., Rafailovich M. (2011). Coping with antibiotic resistance: Combining nanoparticles with antibiotics and other antimicrobial agents. Expert Rev. Anti-Infect. Ther..

[36] Dasari T.S., Zhang Y., Yu H. (2015). Antibacterial activity and cytotoxicity of gold (I) and (III) ions and gold nanoparticles. Biochem. Pharmacol. Open Access.

[37] Rezanejad M., Karimi S., Momtaz H. (2019). Phenotypic and molecular characterization of antimicrobial resistance in Trueperella pyogenes strains isolated from bovine mastitis and metritis. BMC Microbiol..

[38] Ema M., Okuda H., Gamo M., Honda K.A. (2017). A review of reproductive and developmental toxicity of silver nanoparticles in laboratory animals. Reprod. Toxicol..

[39] Al-Bishri W.M. (2018). Toxicity study of gold and silver nanoparticles on experimental animals. Pharmacophore.

[40] Almeida J.C., Cardoso C.E., Pereira E., Freitas R. (2019). Toxic effects of metal nanoparticles in marine invertebrates. Nanostructured Materials for Treating Aquatic Pollution.

